# Treatment of Sciatica

**Published:** 1914-02-28

**Authors:** John J. Grace

**Affiliations:** 61 Welbeck Street, Cavendish Square, W.


### THE EDITOR'S LETTER-BOX.

### Treatment of Sciatica.

*To the Editor of* The Hospital.

Sir,—I see in last week's "Modern Methods" series you refer to the treatment of sciatica by radiant heat and static electricity.

The method originated, I believe, or at least was perfected by Dr. William Benham Snow, of New York, who tells me that he has treated upwards of four hundred cases of neuritis, including seventy cases of sciatica, by this method. He adds that all the early cases of sciatica and a large proportion of all cases were relieved.

Recently I reported in the *Lancet* nine cases of sciatica treated by me by this method, eight of which were relieved. As the ninth showed no improvement after about twenty treatments, and also no improvement after treatment by the $x$-ray by the method described by Delheim and Pz in the *Archives of the Röntgen Ray*, March 1913, I took a skiagram, which showed evidence of osteo-arthritis of the hip-joint. I therefore discontinued treatment, as I considered it probable that the pain was due to the osteo-arthritis. I have communicated with the other eight patients, and find that in all cases except one they remain symptomatically cured. This last patient left me to take a holiday while he still had some stiffness in the limb. I told him to return after his holiday, as I did not consider him well. However, he did not do so until the pain returned. He is now again recovering rapidly. This patient has remained at his very arduous physical work during the whole course of the treatment. Ten days ago I began the treatment of a man who has had sciatica since September last. He now has no pain to speak of, but still has a stiff back. He has returned to work at a job which entails a good deal of weight-lifting. Whether he will be able to continue at it remains to be seen. It is fair to add that he was already improving when he came to me.

Shortly, the treatment consists in exposing the part to the rays of a 500-c.p. single-filament lamp as hot as can be borne for twenty to thirty minutes. After this the static wave current is applied to the part through a flexible metal electrode covering the area from below the trochanter to the sacro-iliac joint for another twenty minutes, and afterwards applying static sparks to tender points, muscles in a state of spasm, and to the course of the sciatic nerve. Occasionally the inflamed area of the nerve can only be reached with a rectal electrode, and in that case the static wave current is administered by that means.

The wave current can be painless or very painful according as it is applied. A person who has no sciatica or fibrositis will easily bear a spark gap of a length of one foot, passing between the prime conductors, when applied with an electrode measuring eight inches by four, while a person with acute sciatica will complain loudly of a spark gap of an inch or less with the same electrode. No advantage in the result is obtained by causing severe pain—indeed, I am not sure that it is not absolutely detrimental to do so. Static sparks are absolutely disagreeable to some people. Others do not appear to mind them. While they are not essential, I think there is no doubt but that those who submit to them get well more quickly.

It is not difficult to understand the action of the static wave current if the effects be watched in, say, a case of recent sprained ankle. Here with one treatment the swelling is visibly reduced, and the patient's comfort correspondingly increased. It is obvious that exuded lymph is returned to its proper channels. So also is the treatment in callous ulcer; the indurated margin gets softer and gradually melts down to the level of the floor, and early and lasting healing is brought about. In sciatica we cannot see what is taking place, but doubtless the process is the same.

Dr. Wm. Bruce has recently published a very interesting little book, embodying his conclusions drawn from an experience of nearly 700 cases of sciatica. He concludes that sciatica is a symptom of disease of the hip-joint. No one who has seen many cases of sub-acute or chronic sciatica can fail to be impressed with the difficulty of diagnosis between this complaint and rheumatoid arthritis. Even with the aid of the $x$-ray, valuable as it is, it is impossible sometimes to decide which condition we are dealing with, for some early undoubted cases of rheumatoid arthritis in other joints give no certain $x$-ray signs. Nevertheless, I do not think that the evidence is complete that these two diseases are one and the same. It is not rare to see cases of considerable destruction of the hip-joint, in which the patient complains not chiefly or, indeed, seriously of pain, but rather of the growing weakness and uselessness of the limb, while in sciatica it is pain, always pain, that is complained of.

If Dr. Bruce's contention is correct, then we must conclude that rheumatoid arthritis of the hip is curable in the early stages.—I am, &c.,

John J. Grace.

61 Welbeck Street,
Cavendish Square, W.

